# Supplementary material for: The Effects of Orthoptic Therapy on the Surgical Outcome in Children with Intermittent Exotropia: Randomised Controlled Clinical Trial
Source: J Clin Med. 2023 Feb 6;12(4):1283. doi: 10.3390/jcm12041283 (PMC9964836; doi:10.3390/jcm12041283)
Supplement: Supplementary file 1 [file jcm-12-01283-s001.zip › jcm-2085566-supplementary.pdf]

Supplementary Table S1. The criteria for intermittent exotropia.

	The criteria for intermittent exotropia
1	Intermittent OR constant exotropia at distance and either intermittent exotropia or exophoria at near
2	Exo-deviation ≥ 20 PD at near or at distance
3	Evidence of progressive loss of stereoacuity
4	Exotropia present greater than or equal to 50% of waking hours, or weaker control of exodeviation (fusional control score ≥ 3 at near or distance)
5	The appearance of the exotropia is causing psychological problems of patients and parents
6	Best corrected visual acuity (BCVA) in the worse eye is 0.1 logMAR or better and children with amblyopia received a period of treatment
7	Wear appropriate refractive correction spectacles for at least 1 month before surgery
8	No atropine was used within the previous month

PD: prism diopter.
